# Phase I study of Y101D, a bispecific antibody targeting PD-L1 and TGF-β in patients with advanced solid tumors

**DOI:** 10.1093/oncolo/oyag133

**Published:** 2026-04-08

**Authors:** Haishuang Sun, Gang Chen, Jinhui Xue, Yaxiong Zhang, Yuxiang Ma, Yunpeng Yang, Wenfeng Fang, Yuanyuan Zhao, Yan Huang, Shaodong Hong, Ting Zhou, Shen Zhao, Huaqiang Zhou, Xi Chen, Jing Li, Li Zhang, Hongyun Zhao

**Affiliations:** Department of Medical Oncology, State Key Laboratory of Oncology in South China, Guangdong Key Laboratory of Nasopharyngeal Carcinoma Diagnosis and Therapy, Guangdong Provincial Clinical Research Center for Cancer, Sun Yat-sen University Cancer Center, Guangzhou 510060, China; Department of Medical Oncology, State Key Laboratory of Oncology in South China, Guangdong Key Laboratory of Nasopharyngeal Carcinoma Diagnosis and Therapy, Guangdong Provincial Clinical Research Center for Cancer, Sun Yat-sen University Cancer Center, Guangzhou 510060, China; Department of Clinical Research, State Key Laboratory of Oncology in South China, Guangdong Key Laboratory of Nasopharyngeal Carcinoma Diagnosis and Therapy, Guangdong Provincial Clinical Research Center for Cancer, Sun Yat-sen University Cancer Center, Guangzhou, Guangdong 510060, China; Department of Medical Oncology, State Key Laboratory of Oncology in South China, Guangdong Key Laboratory of Nasopharyngeal Carcinoma Diagnosis and Therapy, Guangdong Provincial Clinical Research Center for Cancer, Sun Yat-sen University Cancer Center, Guangzhou 510060, China; Department of Clinical Research, State Key Laboratory of Oncology in South China, Guangdong Key Laboratory of Nasopharyngeal Carcinoma Diagnosis and Therapy, Guangdong Provincial Clinical Research Center for Cancer, Sun Yat-sen University Cancer Center, Guangzhou, Guangdong 510060, China; Department of Medical Oncology, State Key Laboratory of Oncology in South China, Guangdong Key Laboratory of Nasopharyngeal Carcinoma Diagnosis and Therapy, Guangdong Provincial Clinical Research Center for Cancer, Sun Yat-sen University Cancer Center, Guangzhou 510060, China; Department of Medical Oncology, State Key Laboratory of Oncology in South China, Guangdong Key Laboratory of Nasopharyngeal Carcinoma Diagnosis and Therapy, Guangdong Provincial Clinical Research Center for Cancer, Sun Yat-sen University Cancer Center, Guangzhou 510060, China; Department of Medical Oncology, State Key Laboratory of Oncology in South China, Guangdong Key Laboratory of Nasopharyngeal Carcinoma Diagnosis and Therapy, Guangdong Provincial Clinical Research Center for Cancer, Sun Yat-sen University Cancer Center, Guangzhou 510060, China; Department of Medical Oncology, State Key Laboratory of Oncology in South China, Guangdong Key Laboratory of Nasopharyngeal Carcinoma Diagnosis and Therapy, Guangdong Provincial Clinical Research Center for Cancer, Sun Yat-sen University Cancer Center, Guangzhou 510060, China; Department of Medical Oncology, State Key Laboratory of Oncology in South China, Guangdong Key Laboratory of Nasopharyngeal Carcinoma Diagnosis and Therapy, Guangdong Provincial Clinical Research Center for Cancer, Sun Yat-sen University Cancer Center, Guangzhou 510060, China; Department of Medical Oncology, State Key Laboratory of Oncology in South China, Guangdong Key Laboratory of Nasopharyngeal Carcinoma Diagnosis and Therapy, Guangdong Provincial Clinical Research Center for Cancer, Sun Yat-sen University Cancer Center, Guangzhou 510060, China; Department of Medical Oncology, State Key Laboratory of Oncology in South China, Guangdong Key Laboratory of Nasopharyngeal Carcinoma Diagnosis and Therapy, Guangdong Provincial Clinical Research Center for Cancer, Sun Yat-sen University Cancer Center, Guangzhou 510060, China; Department of Medical Oncology, State Key Laboratory of Oncology in South China, Guangdong Key Laboratory of Nasopharyngeal Carcinoma Diagnosis and Therapy, Guangdong Provincial Clinical Research Center for Cancer, Sun Yat-sen University Cancer Center, Guangzhou 510060, China; Department of Medical Oncology, State Key Laboratory of Oncology in South China, Guangdong Key Laboratory of Nasopharyngeal Carcinoma Diagnosis and Therapy, Guangdong Provincial Clinical Research Center for Cancer, Sun Yat-sen University Cancer Center, Guangzhou 510060, China; Department of Medical Oncology, State Key Laboratory of Oncology in South China, Guangdong Key Laboratory of Nasopharyngeal Carcinoma Diagnosis and Therapy, Guangdong Provincial Clinical Research Center for Cancer, Sun Yat-sen University Cancer Center, Guangzhou 510060, China; Department of Medical Oncology, State Key Laboratory of Oncology in South China, Guangdong Key Laboratory of Nasopharyngeal Carcinoma Diagnosis and Therapy, Guangdong Provincial Clinical Research Center for Cancer, Sun Yat-sen University Cancer Center, Guangzhou 510060, China; Department of Clinical Research, State Key Laboratory of Oncology in South China, Guangdong Key Laboratory of Nasopharyngeal Carcinoma Diagnosis and Therapy, Guangdong Provincial Clinical Research Center for Cancer, Sun Yat-sen University Cancer Center, Guangzhou, Guangdong 510060, China

**Keywords:** Y101D, PD-L1, TGF-β, tumor, immunotherapy

## Abstract

**Background:**

Y101D is a novel bispecific antibody targeting PD-L1 and TGF-β. This multicenter phase I study evaluated the safety, tolerability, pharmacokinetics, and pharmacodynamics of Y101D in patients with metastatic or locally advanced solid tumors.

**Methods:**

Patients who failed standard therapies were enrolled. In the dose-escalation and -expansion phases, Y101D was administered intravenously every 2 weeks (Q2W) at 1, 3, 10, 20, and 30 mg/kg. Primary objectives were dose-limiting toxicities (DLTs) and maximum tolerated dose (MTD) in escalation, safety, and objective response rate (ORR) in expansion. Secondary objectives included pharmacokinetics/pharmacodynamics parameters, and immunogenicity.

**Results:**

Among 50 enrolled patients, the most common treatment-related adverse events (TRAEs) were aspartate aminotransferase elevation (20.0%), gingival bleeding (18.0%), alanine aminotransferase elevation (14.0%), rash (14.0%), and proteinuria (14.0%). Grade ≥ 3 TRAEs occurred in 10.0% of patients, with no grade 4/5 TRAEs. Immune-related adverse events (irAEs) occurred in 22.0%, predominantly grades 1-2. No DLTs were observed, and MTD was not reached. One extensive-stage small cell lung cancer (ES-SCLC) patient (20 mg/kg Q2W) achieved a confirmed partial response (ORR: 2.1%, 95% CI, 0.1%-11.3%). Median progression-free survival and overall survival were 1.3 months (95% CI, 0.9-1.3) and 10.5 months (95% CI, 6.6-12.7), respectively. The pharmacokinetic characteristics of single and multiple doses of 20 mg/kg Q3W and 1200 mg Q3W supported the administration schedule of once every 3 weeks. PD-L1 target occupancy exceeded 95% at 2 h post-dose across all cohorts and remained sustained pre-dose.

**Conclusions:**

Y101D monotherapy exhibited a manageable safety profile and on-target pharmacodynamic activity, with limited antitumor activity as a single agent. Further evaluation in disease-focused cohorts and rational combination regimens is warranted, including in ES-SCLC.

**Clinical Trial number registration:**

NCT05028556

Lessons Learned

- Y101D monotherapy demonstrated a manageable safety profile with no observed DLTs and no grade 4/5 TRAEs.
- Y101D achieved rapid and sustained on-target pharmacodynamic activity (PD-L1 target occupancy >95% and suppression of circulating free TGF-β isoforms).
- Antitumor activity was limited in this heterogeneous, heavily pretreated phase I population, supporting disease-focused evaluation and rational combinations.

## Trial information

**Table:** 

**TRIAL INFORMATION**	
**Disease**	Advanced or metastatic solid tumors [including ES-SCLC, microsatellite stable (MSS)/proficient mismatch repair (pMMR) colorectal cancer, biliary tract cancer]
**Stage of disease/treatment**	Advanced/metastatic
**Prior therapy**	Failed standard therapies; heavily pretreated
**Type of study**	Multicenter, open-label, phase I dose-escalation, and dose-expansion
**Primary endpoints**	DLTs and MTD (escalation); safety and ORR (expansion)
**Secondary endpoints**	Pharmacokinetics (PK)/pharmacodynamics (PD), immunogenicity, PFS, OS, disease control rate (DCR)
**Additional details of endpoints or study design**	3 + 3 escalation; every 2 weeks (Q2W) dosing at 1, 3, 10, 20, 30 mg/kg; expansion included Q2W and every 3 weeks (Q3W) regimens. Tumor response assessed per RECIST v1.1 (or iRECIST when applicable).

## Drug information

**Table:** 

**DRUG INFORMATION**	
**Generic/Working name**	Y101D
**Company name**	Wuhan YZY Biopharma Co., Ltd.
**Drug type**	Bispecific antibody
**Drug class**	PD-L1 and TGF-β dual-targeting immunotherapy
**Dose**	Dose-escalation: 1, 3, 10, 20, 30 mg/kg Q2W; Expansion included 20 mg/kg Q2W, 20 mg/kg Q3W, and 1200 mg Q3W
**Route**	Intravenous infusion
**Schedule of administration**	Q2W or Q3W depending on cohort

## Dose-escalation table

**Table:** 

**DOSE-ESCALATION TABLE**				
Dose level	Dose of drug	Schedule	Number enrolled	Number evaluable for toxicity
**1**	1 mg/kg	Q2W	3	3
**2**	3 mg/kg	Q2W	3	3
**3**	10 mg/kg	Q2W	3	3
**4**	20 mg/kg	Q2W	9	9
**5**	30 mg/kg	Q2W	9	9
**Expansion**	20 mg/kg	Q3W	12	12
**Expansion**	1200 mg	Q3W	11	11

## Patient characteristics

Fifty patients were enrolled between August 2021 and January 2024. Median age was 55.5 years (range across cohorts approximately 44-67.5), and 34 (68.0%) were male. Tumor types included SCLC (*n* = 18, 36.0%), colorectal cancer (*n* = 10, 20.0%), cholangiocarcinoma (*n* = 4, 8.0%), pulmonary lymphoepithelioma-like carcinoma (*n* = 3, 6.0%), lung adenocarcinoma (*n* = 3, 6.0%), and other malignancies (Table 1).

### Patient characteristics table

**Table:** 

**PATIENT CHARACTERISTICS**	
**Number of patients, male**	34 (68.0)
**Number of patients, female**	16 (32.0)
**Stage**	III-IV
**Performance status: ECOG 0 or 1**	50 (100.0)
**Performance status: ECOG 2 or above**	0
**Cancer types or histologic subtypes**	SCLC 18 (36.0); CRC 10 (20.0); cholangiocarcinoma 4 (8.0); others 18 (36.0)

### Primary assessment method

**Table:** 

**PRIMARY ASSESSMENT METHOD**	
**Title**	Safety and antitumor activity of Y101D
**Number of patients screened**	81
**Number of patients enrolled**	50
**Number of patients evaluable for toxicity**	50
**Number of patients evaluated for efficacy**	47
**Evaluation method**	Safety: CTCAE v5.0; Response: RECIST v1.1 (or iRECIST); PFS/OS: Kaplan–Meier

### Serious adverse events table

**Table:** 

SERIOUS ADVERSE EVENTS		
Dose Level	Adverse Event(s), Grade	Attribution
**1 mg/kg Q2W**	Infectious pneumonia, gr 1	Unlikely
**10 mg/kg Q2W**	Pulmonary infection, gr 3	Unrelated
**10 mg/kg Q2W**	Hemoptysis (worsened), gr 2	Related
**20 mg/kg Q2W**	Fever, gr 2; Hemoptysis, gr 2; Hyponatremia, gr 4	Possible/Unlikely
**20 mg/kg Q2W**	Infectious pneumonia, gr 3	Unlikely
**20 mg/kg Q2W**	Lymphadenitis, gr 3	Unlikely
**20 mg/kg Q2W**	Liver function abnormality, gr 3 (2 events); Immune-mediated diabetes mellitus, gr 3	Probable/Related
**30 mg/kg Q2W**	Hemoptysis, gr 2; Epistaxis, gr 2	Unlikely
**30 mg/kg Q2W**	Epistaxis, gr 3	Possible
**30 mg/kg Q2W**	Infectious pneumonia, gr 3	Unlikely
**20 mg/kg Q3W**	Infectious pneumonia (death), gr 5	Unrelated
**20 mg/kg Q3W**	Intestinal obstruction, gr 3	Unrelated
**1200 mg Q3W**	Hemoptysis, gr 2	Unlikely

## General toxicity profile

The median duration of drug exposure was 17 days (range: 15-323) for 20 mg/kg Q2W, 25 days (22-94) for 20 mg/kg Q3W, and 22 days (1-63) for 1200 mg Q3W. In the safety set (*n* = 50), 49 participants (98.0%) experienced AEs and 35 (70.0%) experienced treatment-related AEs (TRAEs). Grade ≥ 3 TRAEs occurred in five participants (10.0%); no grade 4-5 TRAEs and no protocol-defined adverse events of special interest were observed. Bleeding events were among the common TRAEs, bleeding-related TRAEs predominantly involved mucosal and urinary tract sites, including gingival bleeding (18.0%), hemoptysis (12.0%), epistaxis (6.0%), and hematuria (6.0%); most bleeding events were grade 1-2 (Table 2). Management was primarily supportive and symptomatic, with temporary treatment interruption implemented when clinically indicated for higher-grade events. Given the advanced disease setting, baseline patient- and tumor-related factors that may predispose to bleeding (eg, tumor involvement of airway or mucosa, prior local therapies, concomitant medications, or coagulation abnormalities) could have contributed to the observed bleeding profile in this small phase I cohort. Treatment-related irAEs demonstrated a dose-dependent trend, with no events observed at doses below 10 mg/kg Q2W. The incidence was 55.6% (5/9) in the 20 mg/kg Q2W cohort, 22.2% (2/9) in the 30 mg/kg Q2W cohort, 8.3% (1/12) in the 20 mg/kg Q3W cohort, and 27.3% (3/11) in the 1200 mg Q3W cohort. Given the limited sample size, conclusions regarding dose–toxicity relationships for irAEs are descriptive and not definitive. Events in the 20 mg/kg Q2W group included hypothyroidism, pruritus, and rash; the 30 mg/kg Q2W group reported pyrexia, myositis, and myocarditis; while the 1200 mg Q3W group primarily featured hepatic enzyme elevations. All irAEs were managed according to institutional guidelines, which included symptomatic treatment, corticosteroids, and/or hormone replacement therapy as clinically indicated. Dose modifications or interruptions were implemented based on the severity of the event. Dose interruptions due to treatment-related grade ≥ 3 AEs were documented in four patients: in the 20 mg/kg Q2W cohort, one patient had an interruption for hemoptysis, and one patient had an interruption for liver function abnormality and diabetes mellitus; in the 30 mg/kg Q2W cohort, one patient had an interruption for epistaxis; and in the 1200 mg Q3W cohort, one patient had an interruption for epistaxis. One patient discontinued due to intestinal obstruction, one died from infectious pneumonia, and one withdrew; no TRAEs led to permanent discontinuation, death, or withdrawal. Dose reductions were not planned; delays/interruptions were permitted. No DLTs occurred during D1-D28, and the MTD was not reached.

**Table 1:** Patient baseline characteristics grouped by dose level.

Characteristics	Q2W-1 mg/kg (*N* = 3)	Q2W-3 mg/kg (*N* = 3)	Q2W-10 mg/kg (*N* = 3)	Q2W-20 mg/kg (*N* = 9)	Q2W-30 mg/kg (*N* = 9)	Q3W-20 mg/kg (*N* = 12)	Q3W-1200 mg (*N* = 11)	Total (*N* = 50)
**Age, median (range), years**	60.0 (57.0, 64.0)	53.0 (52.0, 63.0)	59.0 (54.0, 66.0)	52.0 (50.0, 56.0)	55.0 (51.0, 59.0)	58.0 (49.0, 67.5)	53.0 (44.0, 59.0)	55.5 (51.0, 60.0)
**Sex, *n* (%)**								
** Male**	2 (66.7)	1 (33.3)	2 (66.7)	7 (77.8)	5 (55.6)	8 (66.7)	9 (81.8)	34 (68.0)
** Female**	1 (33.3)	2 (66.7)	1 (33.3)	2 (22.2)	4 (44.4)	4 (33.3)	2 (18.2)	16 (32.0)
**ECOG performance status *n* (%)**								
** 0**	0	1 (33.3)	1 (33.3)	0	0	0	0	2 (4.0)
** 1**	3 (100)	2 (66.7)	2 (66.7)	9 (100)	9 (100)	12 (100)	11 (100)	48 (96.0)
**Diagnosis, *n* (%)**								
** Nasopharyngeal carcinoma**	0	0	0	0	2 (22.2)	0	0	2 (4.0)
** Cholangiocarcinoma**	0	0	0	0	0	2 (16.7)	2 (18.2)	4 (8.0)
** Malignant mesothelioma**	0	1 (33.3)	0	1 (11.1)	0	0	0	2 (4.0)
** Pulmonary squamous cell carcinoma**	1 (33.3)	0	1 (33.3)	0	0	0	0	2 (4.0)
** Pulmonary lymphoepithelioma-like carcinoma**	0	2 (66.7)	0	0	1 (11.1)	0	0	3 (6.0)
** Pulmonary adenocarcinoma**	2 (66.7)	0	0	0	1 (11.1)	0	0	3 (6.0)
** Hepatocellular carcinoma**	0	0	0	0	1 (11.1)	0	0	1 (2.0)
** Cervical carcinoma**	0	0	1 (33.3)	0	0	0	0	1 (2.0)
** Melanoma**	0	0	0	0	1 (11.1)	0	0	1 (2.0)
** Colorectal carcinoma**	0	0	0	3 (33.3)	1 (11.1)	6 (50.0)	0	10 (20.0)
** Esophageal carcinoma**	0	0	0	0	1 (11.1)	0	0	1 (2.0)
** Small-cell lung carcinoma**	0	0	0	5 (55.6)	0	4 (33.3)	9 (81.8%)	18 (36.0)
** Pancreatic carcinoma**	0	0	1 (33.3)	0	0	0	0	1 (2.0)
** Endometrial carcinoma**	0	0	0	0	1 (11.1)	0	0	1 (2.0)

Abbreviations: ECOG, Eastern Cooperative Oncology Group; Q2W, every 2 weeks; Q3W, every 3 weeks.

**Table 2:** TRAE grouped by dose level.

	Q2W-1 mg/kg (*N* = 3)	Q2W-3 mg/kg (*N* = 3)	Q2W-10 mg/kg (*N* = 3)	Q2W-20 mg/kg (*N* = 9)	Q2W-30 mg/kg (*N* = 9)	Q3W-20 mg/kg (*N* = 12)	Q3W-1200 mg (*N* = 11)	Total (*N* = 50)
**Any TRAE**	1 (33.3)	2 (66.7)	2 (66.7)	7 (77.8)	8 (88.9)	7 (58.3)	8 (72.7)	35 (70.0)
**SAE**	0	0	0	2 (22.2)	2 (22.2)	0	1 (9.1)	5 (10.0)
**irAE**	0	0	0	5 (55.6)	2 (22.2)	1 (8.3)	3 (27.3)	11 (22.0)
**Most common TRAE (≥5% of the total population)**	0	1 (33.3)	2 (66.7)	5 (55.6)	3 (33.3)	2 (16.7)	7 (63.6)	20 (40.0)
** AST**	0	0	0	5 (55.6)	1 (11.1)	0	4 (36.4)	10 (20.0)
** ALT**	0	0	0	4 (44.4)	0	0	3 (27.3)	7 (14.0)
** Alkaline phosphatase increased**	0	0	0	2 (22.2)	1 (11.1)	0	1 (9.1)	4 (8.0)
** Lipase increased**	0	1 (33.3)	0	0	0	1 (8.3)	1 (9.1)	3 (6.0)
** GGT increased**	0	0	0	2 (22.2)	1 (11.1)	0	0	3 (6.0)
** Platelet count decreased**	0	0	1 (33.3)	1 (11.1)	1 (11.1)	0	0	3 (6.0)
** Bilirubin increased**	0	0	0	0	0	0	3 (27.3)	3 (6.0)
** Gingival bleeding**	0	2 (66.7)	0	2 (22.2)	1 (11.1)	3 (25.0)	1 (9.1)	9 (18.0)
** Nausea**	0	0	0	0	0	2 (16.7)	2 (18.2)	4 (8.0)
** Vomiting**	0	0	0	1 (11.1)	0	1 (8.3)	1 (9.1)	3 (6.0)
** Fever**	0	0	0	0	2 (22.2)	2 (16.7)	1 (9.1)	5 (10.0)
** Fatigue**	0	0	0	1 (11.1)	2 (22.2)	1 (8.3)	0	4 (8.0)
** Rash**	1 (33.3)	0	0	1 (11.1)	2 (22.2)	3 (25.0)	0	7 (14.0)
** Hemoptysis**	0	0	0	2 (22.2)	1 (11.1)	1 (8.3)	2 (18.2)	6 (12.0)
** Epistaxis**	0	0	0	1 (11.1)	2 (22.2)	0	0	3 (6.0)
** Proteinuria**	0	0	1 (33.3)	3 (33.3)	2 (22.2)	1 (8.3)	0	7 (14.0)
** Hematuria**	0	0	0	1 (11.1)	1 (11.1)	1 (8.3)	0	3 (6.0)
** Hyponatremia**	0	0	0	2 (22.2)	0	0	1 (9.1)	3 (6.0)
** Appetite decreased**	0	0	0	1 (11.1)	0	0	2 (18.2)	3 (6.0)
** Anemia**	0	0	1 (33.3)	2 (22.2)	2 (22.2)	0	1 (9.1)	6 (12.0)
**Grade ≥3 TRAE**	0	0	0	2 (22.2)	2 (22.2)	0	1 (9.1)	5 (10.0)
** Hemoptysis**	0	0	0	1 (11.1)	0	0	1 (9.1)	2 (4.0)
** Epistaxis**	0	0	0	0	1 (11.1)	0	0	1 (2.0)
** GGT increased**	0	0	0	1 (11.1)	1 (11.1)	0	0	2 (4.0)
** Liver function abnormal**	0	0	0	1 (11.1)	0	0	0	1 (2.0)
** Type 1 diabetes mellitus**	0	0	0	1 (11.1)	0	0	0	1 (2.0)

Abbreviations: ALT, alanine aminotransferase; AST, aspartate aminotransferase; GGT, gamma-glutamyl transferase; irAE, immune-related adverse event; Q2W, every 2 weeks; Q3W, every 3 weeks; SAE, serious adverse event; TRAE, treatment-related adverse event.

### Dose-limiting toxicities

**Table:** 

DOSE-LIMITING TOXICITIES			
Dose level	Number evaluable	DLT observed (Yes/No)	DLT description
**All evaluated dose levels**	15 (dose-escalation) + expansion cohorts	No	No protocol-defined DLTs occurred during D1-D28; MTD not reached.

## Pharmacokinetics & pharmacodynamics

**Figure 1.**
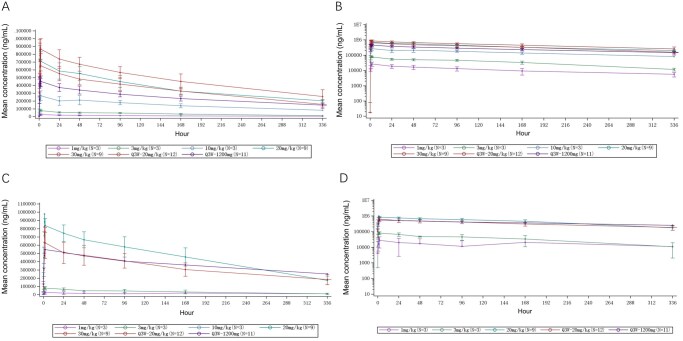
Mean plasma concentration–time curves of Y101D stratified by dose level following (A) a single administration on a linear scale, (B) a single administration on a semilogarithmic scale, (C) multiple administrations on a linear scale, and (D) multiple administrations on a semilogarithmic scale. Q2W, every 2 weeks; Q3W, every 3 weeks.

In the dose-escalation phase, after a single dose, Cmax increased approximately proportionally with dose, whereas AUC0–t and AUC0–∞ showed a trend toward greater-than-dose-proportional increases (Figure 1A and B), noting the variability and the limited sample size within each dose cohort. Pharmacokinetic parameters are summarized in Table S1.

**Figure 2.**
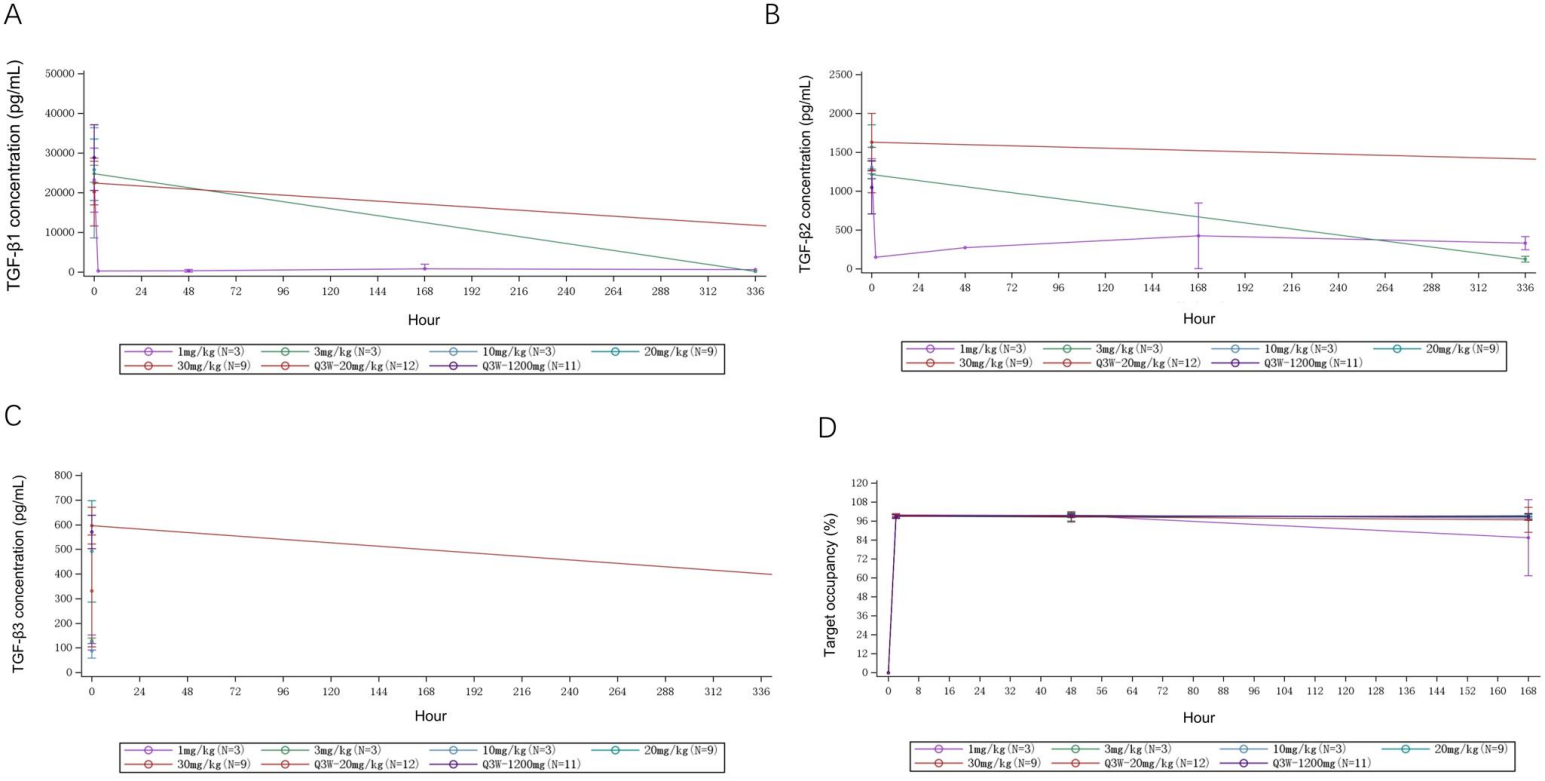
Pharmacodynamic Profiles Following Y101D Treatment. (A) Mean TGF-β1 concentration–time curve; (B) Mean TGF-β2 concentration–time curve; (C) Mean TGF-β3 concentration–time curve; (D) Mean PD-L1 target occupancy–time curve.

Y101D induced rapid and sustained suppression of circulating TGF-β1/2/3 at doses ≥10 mg/kg, with levels reaching the lower limit of quantification within 2 h post-dose, accompanied by sustained PD-L1 target occupancy >95% across all cohorts (Figure 2).

## Efficacy

**Figure 3.**
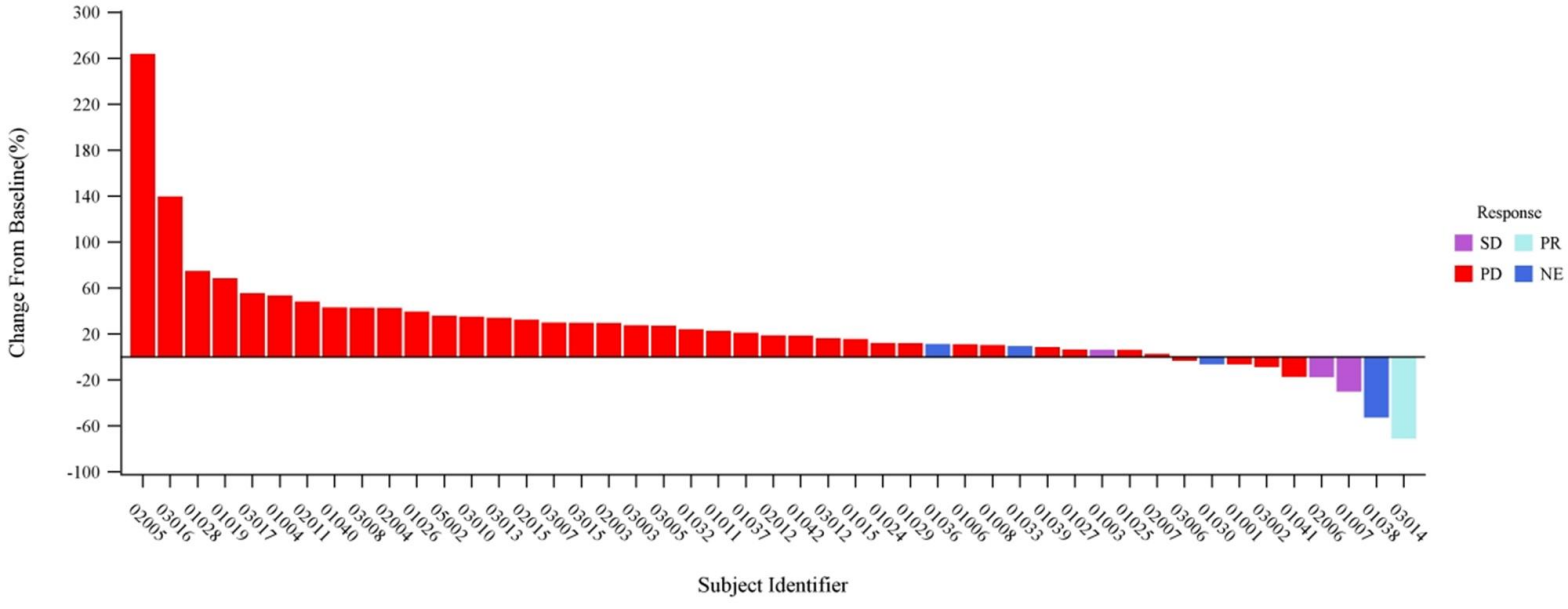
Tumor response to Y101D. Best percentage change from baseline in target lesion size.

At data cutoff (January 15, 2024), 47 patients were evaluable for efficacy. One ES-SCLC patient treated with 20 mg/kg Q2W achieved a confirmed PR (ORR 2.1%, 95% CI, 0.1-11.3), and three patients had SD (DCR 8.5%, 95% CI, 2.4-20.4; Figure 3**)**. Confirmed DCR was observed at 1 mg/kg Q2W (33.3%), 3 mg/kg Q2W (33.3%), and 20 mg/kg Q2W (22.2%), but not at other doses. Median PFS and OS were 1.3 months (95% CI, 0.9-1.3) and 10.5 months (95% CI, 6.6-12.7), respectively (Table S2 and Figure S1). No dose–response relationship was observed, and time-to-event endpoints should be interpreted cautiously given the small, heterogeneous population and potential influence of postprogression therapies. In the ES-SCLC expansion cohort, ORR and DCR were 7.1% and 14.3% **(**Table S3), with median PFS and OS of 1.3 and 11.5 months, respectively (Table S4 and Figure S2). In conclusion, Y101D monotherapy demonstrates limited efficacy in the treatment of metastatic or locally advanced solid tumors. No confirmed ORR were observed in other tumor-type cohorts.

## Discussion

This phase I study demonstrates that Y101D monotherapy has a manageable safety profile and on-target pharmacodynamic activity, with limited antitumor activity as a single agent in a heavily pretreated and heterogeneous population. These findings support further evaluation of Y101D, particularly in disease-focused studies and rational combinations, while recognizing that any potential to address immune-checkpoint resistance requires confirmation in adequately powered trials.

Structurally, Y101D represents an advancement in bispecific antibody engineering. Its predecessor YM101, a first-generation PD-L1/TGF-β fusion protein, was limited by large molecular size and nonselective TGF-β sequestration, leading to suboptimal tumor penetration and potential systemic toxicity.^1^ In contrast, Y101D is built on the Check-BODY platform with a symmetric tetravalent (2:2) (Fab–Fv)_2_–Fc design, improving chain pairing and molecular homogeneity. Its anti-TGF-β arm is an antibody-derived Fv rather than a receptor trap, and Fc modifications eliminate antibody-dependent cellular cytotoxicity (ADCC) and complement-dependent cytotoxicity (CDC) activities, collectively supporting a prolonged half-life, reduced off-target toxicity, and a potentially improved safety profile compared with fusion protein-based agents such as M7824 and receptor-based constructs like SHR-1701.

Y101D demonstrated an acceptable safety profile with no DLTs. Grade ≥ 3 TRAEs occurred in 10% of patients, numerically lower than reported for M7824 (21%) and SHR-1701 (22%).^2^^,^^3^ Common TRAEs (elevated ALT, rash, and proteinuria) were consistent with SHR-1701.^2^ Bleeding-related TRAEs, mainly mucosal and urinary tract events, warrant cautious interpretation given the small, heterogeneous phase I population and baseline bleeding risk factors,^2^^,^^4^ supporting careful monitoring and further characterization in larger, indication-specific cohorts. Notably, TGF-β-associated skin toxicities reported with M7824 (eg, squamous cell carcinoma and keratoacanthoma) were not observed, potentially related to Fc engineering that eliminates ADCC/CDC and reduces off-target immune activation.^5^^,^^6^

Pharmacokinetic analyses showed dose-proportional exposure, a prolonged half-life, sustained PD-L1 occupancy (>95%), and rapid, durable suppression of serum TGF-β1/2/3 across doses. Together with its symmetric tetravalent Check-BODY structure, these findings support Y101D’s molecular stability, manufacturability, and potentially superior safety compared with fusion protein-based or receptor-based constructs such as M7824 and SHR-1701.^7-10^ TGF-β plays a dual role in cancer, acting as a tumor suppressor in early stages but promoting tumor progression and immune evasion in advanced disease,^11^ accordingly, dual PD-(L)1/TGF-β blockade has emerged as a rational strategy to modulate immune exclusion within the tumor microenvironment. However, the magnitude of response varies substantially across studies depending on tumor type, line of therapy, and prior exposure to PD-(L)1 blockade, as well as biomarker enrichment strategies (eg, PD-L1 expression or combined positive score [CPS] thresholds). Bispecific antibodies such as M7824 and SHR-1701, which simultaneously target PD-L1 and TGF-β, have shown encouraging clinical activity with manageable safety profiles. M7824 achieved an ORR of 21.3%, with higher responses in PD-L1-positive (36.0%) and PD-L1 high (≥80% expression on tumor cells) patients (85.7%), though it did not outperform pembrolizumab in a phase 3 NSCLC trial.^12^ SHR-1701 demonstrated efficacy in gastric cancer, with an ORR of 20.0% and a 12-month overall survival rate of 54.5%, particularly in patients with higher PD-L1 CPS scores.^2^ Accordingly, any numerical efficacy comparisons across trials are provided for descriptive context only and should not be construed as evidence of comparative efficacy given heterogeneity in study designs, patient characteristics, and response assessment schedules. In our study, Y101D monotherapy demonstrated limited antitumor activity in an unselected, heavily pretreated population, with one confirmed PR observed in ES-SCLC, suggesting that any apparent activity should be viewed as hypothesis-generating and requires validation in biomarker-enriched, disease-specific cohorts and rational combination strategies. Preclinical data suggest that TGF-β-driven M2 macrophage polarization may contribute to immunotherapy resistance, and dual-targeting antibodies like Y101D may help reshape the tumor immune microenvironment.^13^ Moving forward, biomarker-guided patient selection and larger trials will be key to realizing the full therapeutic potential of this strategy.

Several limitations warrant consideration. The small, heterogeneous sample size and nonrandomized expansion phase limit efficacy analyses to descriptive interpretation rather than evidence of clinical benefit. Time-to-event endpoints may be affected by informative censoring and postprogression therapies, and the observed PFS-OS dissociation should, therefore, be interpreted cautiously in this phase I setting. In addition, biomarker analyses were exploratory and not intended to define prespecified predictive cutoffs or subgroups.

## Conclusions

In summary, while the monotherapy of Y101D in patients with advanced solid tumors demonstrates limited efficacy, the manageable safety profile and pharmacodynamic results provide a foundation for future studies. Ongoing trials exploring combination regimens, particularly in ES-SCLC and other TGF-β-driven tumors, are needed to fully assess the potential of dual PD-L1 and TGF-β inhibition as a therapeutic strategy in oncology.

## Supplementary Material

oyag133_Supplementary_Data

## Data Availability

The datasets generated and analyzed during the study are available from the corresponding author on reasonable request.
