# Supplementary material for: Phase I study of Y101D, a bispecific antibody targeting PD-L1 and TGF-β in patients with advanced solid tumors
Source: Oncologist. 2026 Apr 8;31(6):oyag133. doi: 10.1093/oncolo/oyag133 (PMC13181260; doi:10.1093/oncolo/oyag133)
Supplement: oyag133_Supplementary_Data [file oyag133_supplementary_data.zip › ctr-Supplemental table- clean- R2.docx]

Supplemental Tables

Supplemental Table S1 Pharmacokinetic parameters after a single intravenous infusion of Y101D

| **Pharmacokinetic Parameter**  **(unit)** | **Q2W-1mg/kg**  **(N=3)** | **Q2W-3mg/kg**  **(N=3)** | **Q2W-10mg/kg**  **(N=3)** | **Q2W-20mg/kg**  **(N=9)** | **Q2W-30mg/kg**  **(N=9)** | **Q3W-20mg/kg**  **(N=12)** | **Q3W-1200mg**  **(N=11)** |
| --- | --- | --- | --- | --- | --- | --- | --- |
| T_max_ (h) | 1.98  (1.88,2.00) | 1.03  (1.03,2.00) | 2.00  (2.00,2.00) | 1.38  (1.03,2.00) | 1.97  (1.38,2.00) | 1.63  (1.23,2.00) | 1.30  (1.00,2.02) |
| C_max_(ug/L) | 26,533.3±8759.19(33.01) | 81,466.7±8900.75(10.93) | 268,333.3±62660.46(23.35) | 747,888.9±133079.34(17.79) | 887,222.2±125286.45(14.12) | 684,833.3±155769.55(22.75) | 465,727.3±62504.55(13.42) |
| AUC_0-t_  (h*ug/L) | 3,376,051.673±1648894.5444 | 11,125,948.075±2097462.3544 | 48,677,044.541±8746012.2238 | 116,595,835.593±21276373.7173 | 159,953,335.901±38859945.0210 | 127,054,271.898±30885768.9542 | 92,982,422.495±21416592.7954 |
| AUC_0-∞_  (h*ug/L) | 4,507,092.323±2187520.7231 | 13,393,728.336±2433888.8513 | 68,610,656.754±9311486.1100 | 174,628,988.528±31632292.0231 | 222,383,307.280±55892254.9103 | 162,441,776.783±31197622.1580 | 136,431,049.849±27356436.1036 |
| λ_z_(1/h) | 0.0052±0.0015  (28.85) | 0.0053±0.0009  (17.39) | 0.0040±0.0014  (35.91) | 0.0038±0.0007  (17.60) | 0.0040±0.0013  (32.25) | 0.0049±0.0038  (78.17) | 0.0029±0.0008  (26.95) |
| t_1/2_(h) | 141.909±40.2630  (28.37) | 133.779±23.1778  (17.33) | 190.566±76.6850  (40.24) | 187.837±29.0016  (15.44) | 186.743±47.8410  (25.62) | 195.328±88.8832  (45.50) | 252.661±67.4567  (26.70) |
| AUC__%Extrap_ (%) | 25.679±6.3685 | 16.906±4.2082 | 28.297±14.4933 | 31.331±14.9569 | 26.163±15.6355 | 21.191±16.6114 | 30.997±14.0139 |
| CL (mL/h) | 17.71±7.722  (43.60) | 13.72±3.322  (24.21) | 10.53±1.791  (17.01) | 7.58±2.224  (29.32) | 8.18±2.482  (30.33) | 7.52±1.869  (24.86) | 9.20±2.267  (24.64) |
| V_d_ (mL) | 3381.30±697.010  (20.61) | 2574.33±223.900  (8.70) | 2874.65±1214.254  (42.24) | 1988.37±386.000  (19.41) | 2074.64±419.158  (20.20) | 1964.48±737.487  (37.54) | 3195.50±424.687  (13.29) |

Notes：T_max_ is presented as Median (Min, Max), and other parameters are presented as Mean ± SD (CV%). Abbreviations: PK, pharmacokinetic; T_max_, time to maximum concentration; C_max_, maximum concentration; AUC, area under time-concentration curve; CL, clearance; V_d_, apparent volume of distribution

Abbreviations: Q2W, every 2 weeks; Q3W, every 3 weeks

Supplemental Table S2 Objective response determined by RECIST v1.1

| **Efficacy** | **Q2W-1mg/kg**  **(N=3)** | **Q2W-3mg/kg**  **(N=3)** | **Q2W-10mg/kg**  **(N=3)** | **Q2W-20mg/kg**  **(N=9)** | **Q2W-30mg/kg**  **(N=8)** | **Q3W-20mg/kg**  **(N=12)** | **Q3W-1200mg/kg**  **(N=9)** | **Total**  **(N=47)** |
| --- | --- | --- | --- | --- | --- | --- | --- | --- |
| Best overall response, n (%) |  |  |  |  |  |  |  |  |
| CR | 0 | 0 | 0 | 0 | 0 | 0 | 0 | 0 |
| PR | 0 | 0 | 0 | 1 (11.1) | 0 | 0 | 0 | 1 (2.1) |
| SD | 1 (33.3) | 1 (33.3) | 0 | 1 (11.1) | 0 | 0 | 0 | 3 (6.4) |
| PD | 2 (66.7) | 2 (66.7) | 3 (100) | 7 (77.8%) | 8 (100) | 8 (66.7) | 9 (100) | 39 (83.0) |
| NE | 0 | 0 | 0 | 0 | 0 | 4 (33.3%) | 0 | 4 (8.5) |
| ORR, % (95% CI) | 0 (0, 70.8) | 0 (0, 70.8) | 0 (0, 70.8) | 11.1 (0.3, 48.2) | 0 (0, 36.9) | 0 (0, 26.5) | 0 (0, 33.6) | 2.1 (0.1, 11.3) |
| DCR, % (95% CI) | 33.3 (0.8, 90.6) | 33.3 (0.8, 90.6) | 0 (0, 70.8) | 22.2 (2.8, 60.0) | 0 (0, 36.9) | 0 (0%, 26.5) | 0 (0, 33.6) | 8.5 (2.4, 20.4) |

Abbreviations: CR, complete response; PR, partial response; SD, stable disease; PD, progressive disease; NE, not evaluable; ORR, objective response rate; DCR, disease control rate; CI, confidence interval; Q2W, every 2 weeks; Q3W, every 3 weeks

Supplemental Table S3 Objective response in the expansion phase of the effective dose group by tumor type (efficacy analysis set)

| **Efficacy** | **ES-SCLC (N = 14)** | **Other Tumor Types (N = 13)** |
| --- | --- | --- |
| Best overall response, n (%) |  |  |
| CR | 0 | 0 |
| PR | 1 (7.1) | 0 |
| SD | 1 (7.1) | 0 |
| PD | 11 (78.6) | 10 (76.9) |
| NE | 1 (7.1) | 3 (23.1) |
| ORR, % (95% CI) | 7.1 (0.2, 33.9) | 0.0 (0.0, 24.7) |
| DCR, % (95% CI) | 14.3 (1.8, 42.8) | 0.0 (0.0, 24.7) |

Abbreviations: CR, complete response; PR, partial response; SD, stable disease; PD, progressive disease; NE, not evaluable; ORR, objective response rate; DCR, disease control rate; CI, confidence interval; ES-SCLC, extensive-stage small cell lung cancer.

ORR was defined as the proportion of patients achieving confirmed CR or PR. DCR was defined as the proportion of patients achieving confirmed CR, PR, or SD.

**Supplemental Table S4 Progression-Free Survival and Overall Survival (OS) by Kaplan–Meier Analysis in the Expansion Phase of the Effective Dose Group, by Tumor Type (Efficacy Analysis Set)**

| **Parameter** | **ES-SCLC (N = 14)** | **Other Tumor Types (N = 13)** |
| --- | --- | --- |
| Median PFS, months (95% CI) | 1.3 (1.2, NE) | 1.3 (0.8, 2.6) |
| Median OS, months (95% CI) | 11.5 (5.4, NE) | 12.0 (6.3, NE) |

Abbreviations: PFS, progression-free survival; OS, overall survival; CI, confidence interval; ES-SCLC, extensive-stage small cell lung cancer; NE, not estimable.

Note: Median survival estimates were derived using the Kaplan–Meier method. Upper confidence limits were not estimable in some subgroups due to limited numbers of events at the time of data cutoff.
