# Supplementary material for: Phase I study of Y101D, a bispecific antibody targeting PD-L1 and TGF-β in patients with advanced solid tumors
Source: Oncologist. 2026 Apr 8;31(6):oyag133. doi: 10.1093/oncolo/oyag133 (PMC13181260; doi:10.1093/oncolo/oyag133)

**Supplemental Figures**

**Supplemental Figure S1.** Kaplan–Meier curve for PFS (A) and OS (B) (Efficacy Analysis Set).


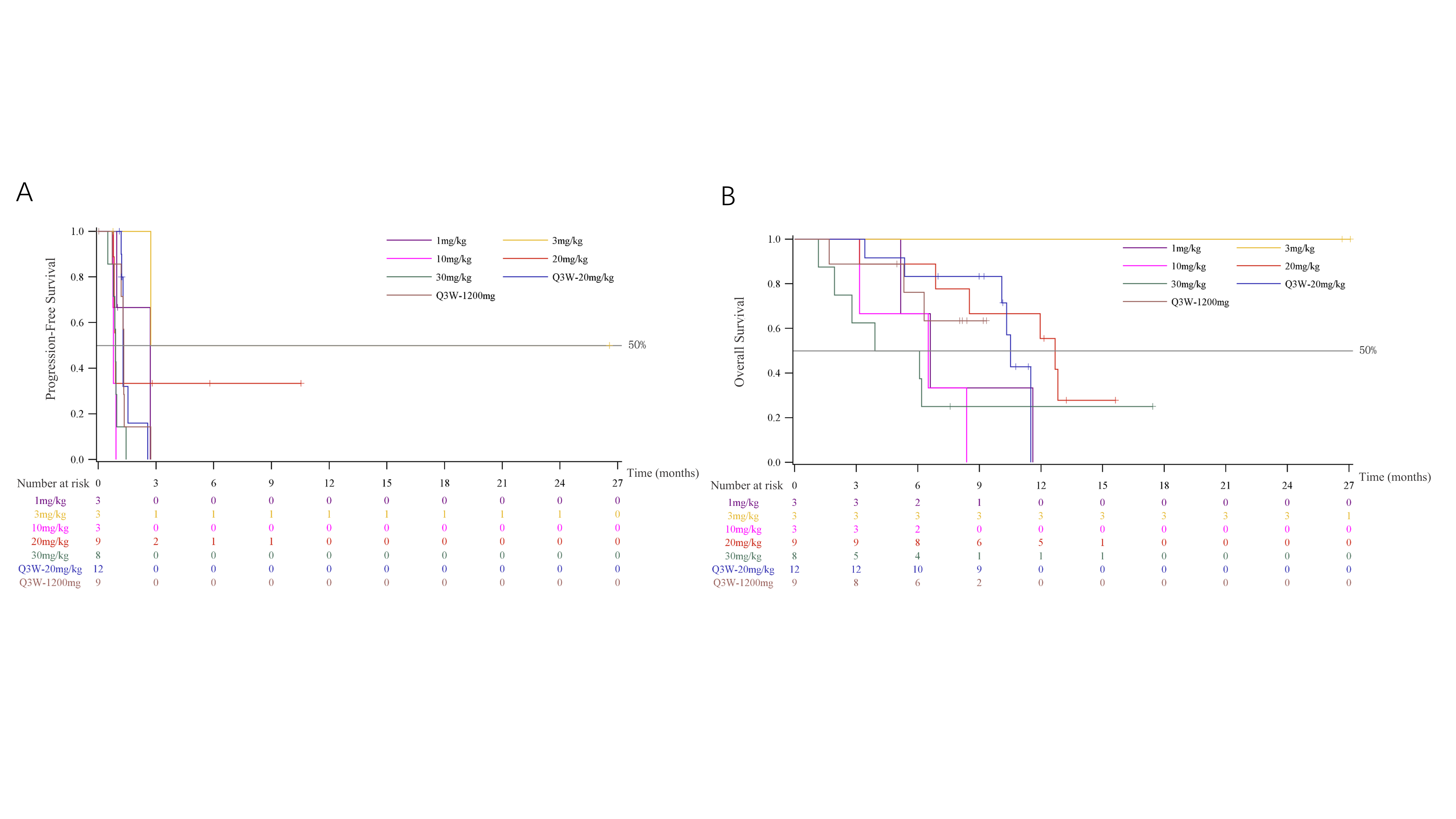


**Supplemental Figure S2.** Kaplan–Meier curve for PFS (A) and OS (B) by tumor type in the expansion phase of the effective dose group.


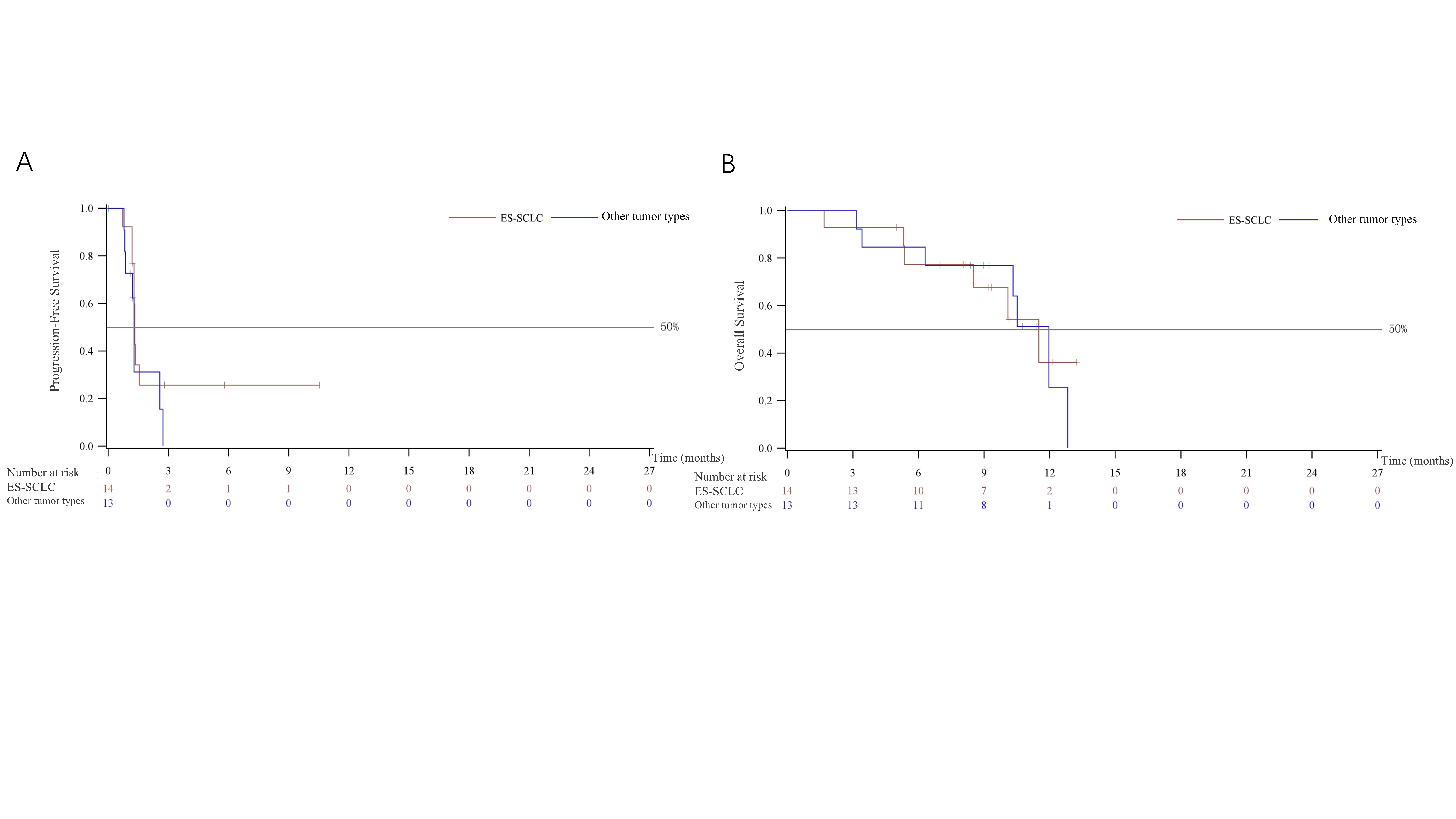

Supplement: oyag133_Supplementary_Data [file oyag133_supplementary_data.zip › ctr-Supplemental figures- clean- R2.docx]
