# Supplementary material for: Phase I study of Y101D, a bispecific antibody targeting PD-L1 and TGF-β in patients with advanced solid tumors
Source: Oncologist. 2026 Apr 8;31(6):oyag133. doi: 10.1093/oncolo/oyag133 (PMC13181260; doi:10.1093/oncolo/oyag133)
Supplement: oyag133_Supplementary_Data [file oyag133_supplementary_data.zip › ctr-Supplemental Methods- clean-R2.docx]

**Supplemental Methods**

**Patients and study design**

This multicenter, open-label, dose-escalation phase I clinical trial enrolled patients with advanced solid tumors. Key inclusion criteria for the dose-escalation and maximum tolerated dose (MTD) expansion phases were: age 18–75 years, histologically confirmed metastatic or locally advanced solid tumors, including treatment-refractory microsatellite stable (MSS)/proficient mismatch repair (pMMR) colorectal cancer, extensive-stage small cell lung cancer (ES-SCLC) after ≥1 prior line of therapy, and biliary tract cancer (intrahepatic cholangiocarcinoma, extrahepatic cholangiocarcinoma, or gallbladder carcinoma) after ≥1 prior line of therapy. Notably, MSS/pMMR colorectal cancer patients were required to be naïve to PD-(L)1 inhibitors. Additional criteria included Eastern Cooperative Oncology Group (ECOG) performance status 0–1, ≥1 measurable lesion per RECIST v1.1, life expectancy ≥12 weeks, and adequate hematologic/hepatic/renal function. Key exclusion criteria comprised: 1) Prior chemotherapy, radiotherapy (except palliative local radiotherapy within 14 days), immunotherapy, or targeted therapy within 28 days before first dose, 2) Previous exposure to PD-(L)1/TGF-β bispecific antibodies, 3) Symptomatic central nervous system metastases requiring intervention, 4) Active autoimmune diseases.

The effective dose expansion phase specifically enrolled patients with MSS/pMMR colorectal cancer, ES-SCLC, or biliary tract cancer. All participants received Y101D monotherapy. The study protocol was approved by institutional ethics committees and conducted in accordance with Good Clinical Practice and the Declaration of Helsinki. Written informed consent was obtained from all participants. Trial registration: ClinicalTrials.gov (NCT05028556).

**Procedures**

The trial comprised dose-escalation and expansion phases. During dose escalation, Y101D was evaluated across five dose levels (1, 3, 10, 20, and 30 mg/kg) administered every 2 weeks (Q2W) according to a standard 3+3 design. Primary objectives included safety evaluation (dose-limiting toxicities [DLTs] and MTD determination), while secondary endpoints encompassed efficacy, immunogenicity, pharmacokinetics, and pharmacodynamics.

Based on integrated safety, pharmacokinetics/pharmacodynamics profiles, and planned combination therapy development, three dose regimens were selected for expansion: 20 mg/kg Q2W, 20 mg/kg Q3W (every 3 weeks), and 1200 mg Q3W. These were evaluated in three cohorts: MSS/pMMR colorectal cancer, ES-SCLC, and biliary tract cancer. The expansion phase primarily assessed safety and ORR, with secondary endpoints including immunogenicity, pharmacokinetics, and pharmacodynamics.

**Pharmacokinetics, immunogenicity, and pharmacodynamics assessments**

Pharmacokinetics analysis, blood samples were collected in both Q2W and Q3W dosing cohorts at multiple timepoints: pre-dose during cycles 1 and 3, and at 30 minutes, 2 hours, 24 hours, 48 hours, 96 hours, and 168 hours post-infusion. An additional sample was collected at 336 hours post-infusion for participants in the Q3W cohort.

For immunogenicity assessment, anti-drug antibody testing was performed at pre-dose during cycles 1 through 4 and at the time of study termination. Pharmacodynamics analyses included evaluation of serum-free TGF-β isoforms (1, 2, and 3) and PD-L1 target occupancy on peripheral blood mononuclear cells. Blood samples for pharmacodynamics analysis were collected at pre-dose, as well as at 2, 48, and 168 hours post-dose in the Q2W cohort, with an additional 336-hour timepoint included for the Q3W cohort.

**Safety and efficacy evaluation**

Safety was evaluated in all treated participants using CTCAE v5.0. Dose-limiting toxicities (DLTs) evaluation window was the first 4 weeks after the initial dosing (D1–D28; W1–W4). Per protocol and CTCAE v5.0, DLT was defined as investigator-attributed, treatment-related toxicity occurring within D1–D28 that met any of the following criteria: (1) any grade ≥4 hematologic toxicity; grade 3 thrombocytopenia accompanied by severe bleeding; or grade 3 febrile neutropenia lasting longer than 1 week; (2) any grade ≥3 non-hematologic toxicity, excluding infusion reaction or allergic reaction and laboratory abnormalities deemed not clinically meaningful, that did not recover to ≤ grade 2 within 3 days despite best supportive care. MTD was determined using the standard 3+3 algorithm: if 0/3 patients experienced DLT, escalation proceeded; if 1/3 experienced DLT, up to 3 additional patients were enrolled at the same dose (n=6); if ≥2/6 experienced DLT, the previous lower dose was defined as the MTD. If the highest prespecified dose level did not meet the MTD threshold, the MTD was considered not reached within the evaluated dose range, and any further escalation or cohort expansion was decided jointly by the investigators and sponsor based on accumulated clinical data. Tumor response was assessed by investigators according to RECIST v1.1 (or iRECIST when applicable) at baseline, at the end of the DLT observation period (Cycle 1), and every 6 weeks thereafter until disease progression. Immune-related adverse events (irAEs) were defined and managed according to institutional guidelines and protocol-specified algorithms.

**Statistical Analysis**

The full analysis set comprised all enrolled participants (n=50). The safety set included all participants who received at least one dose of Y101D (n=50), and the efficacy analysis set included participants with at least one post-baseline tumor assessment (n=47). ORR and DCR were calculated with exact (Clopper–Pearson) 95% confidence intervals. Time-to-event endpoints (PFS, OS, DOR, and TTR) were estimated using the Kaplan–Meier method. Participants without an event at data cutoff were censored at the date of last adequate assessment. Given the heterogeneity of tumor types and the limited sample size, all analyses were descriptive; no formal hypothesis testing or adjustment for multiplicity was performed, and cross-trial (historical control) comparisons were not used. Given the single-arm design and the possibility of informative censoring, Kaplan–Meier estimates should be interpreted as descriptive.
